# The effect of curcumin and high-content eicosapentaenoic acid supplementations in type 2 diabetes mellitus patients: a double-blinded randomized clinical trial

**DOI:** 10.1038/s41387-024-00274-6

**Published:** 2024-04-08

**Authors:** Kimia Motlagh Asghari, Parviz Saleh, Yaghoub Salekzamani, Neda Dolatkhah, Naser Aghamohammadzadeh, Maryam Hashemian

**Affiliations:** 1 Physical Medicine and Rehabilitation Research Center, Tabriz University of Medical Sciences, Tabriz, Iran; 2 Kidney Research Center, Tabriz University of Medical Sciences, Tabriz, Iran; 3 Endocrinology Research Center, Tabriz University of Medical Sciences, Tabriz, Iran; 4 Department of Biology, School of Arts and Sciences, Utica University, Utica, NY USA

**Keywords:** Type 2 diabetes, Enzymes

## Abstract

**Background/objectives:**

The present study investigated the effect of curcumin and eicosapentaenoic acid, as one the main components of omega-3 polyunsaturated fatty acids, on anthropometric, glucose homeostasis, and gene expression markers of cardio-metabolic risk in patients with type 2 diabetes mellitus.

**Subjects/methods:**

This clinical trial was conducted at the Endocrinology Clinic of Imam Reza Hospital in Tabriz. It aimed to determine the impact of Eicosapentaenoic Acid (EPA), Docosahexaenoic Acid (DHA), and curcumin supplements on various health indicators in patients with Type 2 Diabetes Mellitus (DM2) from 2021.02.01 to 2022.02.01. The study was a randomized double-blinded clinical trial and conducted over 12 weeks with 100 participants randomly divided into four groups. Stratified randomization was used to assign participants to two months of supplementation based on sex and Body Mass Index (BMI). The study comprised four groups: Group 1 received 2 capsules of 500 mg EPA and 200 mg DHA, along with 1 nano-curcumin placebo; Group 2 received 1 capsule of 80 mg nano-curcumin and 2 omega 3 Fatty Acids placebos; Group 3 received 2 capsules of 500 mg EPA and 200 mg DHA, and 1 capsule of 80 mg nano-curcumin; Group 4, the control, received 2 omega 3 Fatty Acids placebos and 1 nano-curcumin placebo.

**Results:**

After twelve weeks of taking EPA + Nano-curcumin supplements, the patients experienced a statistically significant reduction in insulin levels in their blood [MD: −1.44 (−2.70, −0.17)]. This decrease was significantly greater than the changes observed in the placebo group [MD: −0.63 (−1.97, 0.69)]. The EPA + Nano-curcumin group also showed a significant decrease in High-Sensitivity C-Reactive Protein (hs-CRP) levels compared to the placebo group (*p* < 0.05). Additionally, the EPA + Nano-curcumin group had a significant increase in Total Antioxidant Capacity (TAC) levels compared to the placebo group (*p* < 0.01). However, there were no significant differences in Fasting Blood Sugar (FBS), Homeostatic Model Assessment of Insulin Resistance (HOMA-IR) index, Quantitative Insulin Sensitivity Check Index (QUICKI), or Hemoglobin A1c (HbA1C) levels between the four groups (all *p* > 0.05). There were significant differences between the Nano-curcumin and EPA groups [MD: −17.02 (−32.99, −1.05)], and between the Nano-curcumin and control groups [MD: −20.76 (−36.73, −4.79)] in terms of lowering the serum cholesterol level. The difference in Triglycerides (TG) serum levels between the EPA + Nano-curcumin and placebo groups were not statistically significant (*p* = 0.093). The Nano-curcumin group showed significant decreases in Low-Density Lipoprotein (LDL) levels compared to the EPA group [MD: −20.12 (−36.90, −3.34)] and the control group [MD: −20.79 (−37.57, −4.01)]. There was a near-to-significant difference in High-Density Lipoprotein (HDL) serum levels between the EPA + Nano-curcumin and EPA groups (*p* = 0.056). Finally, there were significant differences in the decrease of serum Vascular Endothelial Growth Factor (VEGF) levels between the EPA and Nano-curcumin groups [MD: −127.50 (−247.91, −7.09)], the EPA and placebo groups [MD: 126.25 (5.83, 246.66)], the EPA + Nano-curcumin and Nano-curcumin groups [MD: −122.76 (−243.17, −2.35)], and the EPA + Nano- curcumin and placebo groups [MD: 121.50 (1.09, 241.92)].

**Conclusions:**

The findings of the present study suggest that 12-week supplementation with EPA and Nano-curcumin may positively impact inflammation, oxidative stress, and metabolic parameters in patients with diabetes. The supplementation of EPA and Nano-curcumin may be a potential intervention to manage diabetes and reduce the risk of complications associated with diabetes. However, further research is needed to validate the study’s findings and establish the long-term effects of EPA and Nano-curcumin supplementation in patients with diabetes.

## Introduction

Diabetes Mellitus (DM), the most common metabolic disease, is a significant threat to global health [1, 2]. The Global burden and prevalence of DM have been immensely increasing, as has the majority of industrial lifestyles, especially in developing countries, associated with inactivity, excessive calorie intake, and stress [1, 2]. Type 2 DM is characterized by impaired glucose regulation and insulin resistance [3]. DM2 is a complex metabolic disorder characterized by a spectrum of abnormalities known as the ominous octet [4]. This framework includes not only impaired glucose regulation and insulin resistance but also encompasses additional interrelated factors such as impaired incretin hormone action, hepatic glucose overproduction, adipose tissue dysfunction, and altered neural regulation [4, 5]. Acknowledging the ominous octet provides a more nuanced understanding of the multifaceted nature of DM2, emphasizing the interconnected abnormalities contributing to its pathophysiology [5]. The ramifications of DM2 extend far beyond hyperglycemia, encompassing a constellation of interconnected metabolic aberrations, including oxidative stress, dyslipidemia, and compromised vascular health [6, 7]. This multifaceted nature underscores the imperative for comprehensive and innovative therapeutic strategies that transcend traditional approaches [8]. DM2 increases the risk of death from cardiovascular diseases (CVD), especially coronary artery disease, by two to four times. Women with diabetes have four times the risk of CVD death than men [9, 10]. Causative factors of atherosclerosis and CVD in DM are complicated; however, evidence shows that systematic inflammation plays an essential role in its pathogenesis [11, 12]. Oxidative stress, which is a product of an inequity between the reactive oxygen species (ROS) production and the physiological antioxidant defenses, shows a pivotal role in the etiology and advancement of DM2 [13, 14]. Elevated oxidative stress contributes to insulin resistance and also exacerbates vascular dysfunction and lipid dysregulation [13, 15, 16]. Concurrently, disturbances in lipid profiles, including elevated triglycerides (TG) and low- density lipoprotein (LDL) cholesterol, coupled with reduced high-density lipoprotein (HDL) cholesterol, are hallmarks of DM2 and significantly heighten the risk of cardiovascular complications [16–18]. Additionally, VEGF, a vital regulator of vascular health and angiogenesis, has emerged as a pertinent marker in the context of DM2, warranting exploration [19–21]. C-reactive protein (CRP) levels are high in people with cardiovascular conditions and have been considered a significant risk marker for CVD [12, 21], which is a strong predictor for the prognosis of DM2 [22]. Hence, inflammation may partially explain the relationship between CVD and DM.

Among the emerging therapeutic candidates, curcumin, a polyphenolic compound derived from the rhizomes of Curcuma longa (turmeric), and eicosapentaenoic acid (EPA), an omega-3 polyunsaturated fatty acids (PUFAs) predominantly found in marine sources, have captured scientific interest due to their multifaceted pharmacological properties [23]. Both curcumin and EPA have exhibited promising anti-inflammatory, antioxidant, and cardioprotective attributes, which make them attractive contenders for ameliorating the pathophysiological cascades associated with DM2 [24–28]. Omega-3 PUFAs has a vital role in immune and inflammatory mechanism [24] and the progression of atherosclerosis [25]. Due to their anti-inflammatory effects, they also play an indispensable role in preventing CVD, especially in at-risk populations [24]. Fish oil omega-3 PUFAs, i.e., EPA and docosahexaenoic acid (DHA), improve dyslipidemia, vascular function, and platelets and reduce blood pressure [26]. Furthermore, sensitivity to insulin positively have been associated with the attentiveness of omega-3 PUFAs in skeletal muscle [29, 30]. Also, sudden death caused by CVD and arrhythmia are reduced as a response to the consumption of omega-3 PUFAs [26, 31]. In contrast the existing literature presents a mixed picture, with some studies suggesting a protective role of omega-3 PUFAs against atrial fibrillation, while others indicate a potential association with an increased risk [32, 33]. The relationship between omega-3 PUFAs and atrial fibrillation is multifaceted, influenced by factors such as dosage, mechanisms of action, and individual variability [34].

Curcumin, the main active component of turmeric, has potential anti-inflammatory properties [27], reducing the concentration of LDL, hindering platelet aggregation, decreasing the thrombosis risk and myocardial infarction, and regulating blood sugar [28]. Nano-curcumin, employed in our study, is a specialized formulation that utilizes nanotechnology to enhance the bioavailability of curcumin [35]. This innovative approach addresses the inherent challenges associated with curcumin’s low solubility and limited absorption [36]. The nanosized particles in nano-curcumin contribute to increased solubility and improved absorption in the gastrointestinal tract, ultimately leading to enhanced bioavailability [37]. Several studies have demonstrated the superior pharmacokinetic profile of nano-curcumin compared to traditional curcumin supplements [37–39]. These studies consistently report increased blood levels and prolonged retention time, emphasizing the potential of nano-curcumin to exert more pronounced physiological effects [35, 36, 38].

The omega-3 supplement chosen for our study combines EPA and DHA, with a special focus on EPA, to harness the synergistic benefits of these essential fatty acids with a special concentration on EPA. Extensive literature supports the notion that EPA and DHA, two prominent omega-3 PUFAs, play distinct and complementary roles in promoting cardiovascular health and metabolic regulation [40–43]. EPA is known for its anti-inflammatory properties, contributing to the mitigation of systemic inflammation, while DHA is recognized for its role in maintaining membrane integrity and supporting neural function [40]. The combination of EPA and DHA has been associated with improved lipid profiles, insulin sensitivity, and overall cardiovascular health [44, 45]. The decision to prioritize EPA over DHA in our omega-3 fatty acid supplement stems from our targeted focus on cardiovascular and metabolic outcomes. Recent research suggests that EPA, known for its anti-inflammatory properties, may play a pivotal role in modulating inflammatory responses and oxidative stress, critical factors in diabetes pathophysiology [40, 46, 47]. EPA’s unique anti-inflammatory properties have shown promise in influencing markers linked to insulin resistance and dyslipidemia [48]. Recent studies suggest that EPA-centric formulations exhibit anti-inflammatory and antioxidant properties, aligning with our investigation’s focus on inflammation and oxidative stress pathways [49].

Omega-3 PUFAs and curcumin affect several pathological mechanisms and have a relatively high safety profile [50, 51]. They have shown beneficial effects on insulin resistance and DM2 through several means. The combination of curcumin, DHA, and EPA has synergistic anti-inflammatory effects [52–54]. These findings suggest the synergistic anti-inflammatory effects of curcumin with omega-3 fatty acids in treating DM2 [52–54]. Furthermore, these studies mainly focused on DHA as one of the primary omega-3 PUFAs.

Despite the individual merits of curcumin and EPA in addressing these multifaceted aspects of DM2, there exists a dearth of research that investigates their combined effects in a comprehensive manner. The synergy of these bioactive compounds may offer a novel and potent therapeutic approach to mitigate oxidative stress, modulate lipid profiles, and improve vascular health in individuals with DM2. Furthermore, the study of their combined impact on glycemic control, as measured by FBS levels, represents a vital component of DM2 management. The present study aims to evaluate the complementary or synergistic effects of curcumin and omega-3 PUFAs capsules, containing mainly EPA, on anthropometric indices of glucose homeostasis, cardiometabolic risk markers, VEGF gene expression, inflammatory index, and oxidative stress in type 2 diabetes patients. By elucidating the multifaceted effects of these interventions, this research may pave the way for novel therapeutic strategies that offer comprehensive and effective management of DM2, thereby improving the quality of life and reducing the burden of complications in affected individuals.

## Methods

### Study design and setting

This clinical trial was conducted at the Endocrinology Clinic of Imam Reza Hospital in Tabriz. It aimed to determine the impact of EPA, DHA, and curcumin supplements on various health indicators in DM2 patients from 2021.02.01 to 2022.02.01. The study was double-blinded and randomized. Before participation, all patients were acknowledged of the objectives and methods and given written informed consent. Patient information was kept confidential throughout the study. Patients were not charged for their participation and received routine treatments as usual. This study was approved by the Ethics Committee of Tabriz University of Medical Sciences Research (IR.TBZMED.REC.1398.759) and registered on the Iranian Registry of Clinical Trials (RCT20161022030424N5).

### Participants

This study focused on patients with DM2 who were referred to the endocrinology clinic at Imam Reza Hospital. Participants were selected through available sampling, and all patients with diabetes who met the inclusion criteria were involved in the study. In order to be qualified, patients had to be 18 years or older, diagnosed with type 2 DM according to the American Diabetes Association criteria [55], have moderately controlled diabetes (HbA1c > 5.8%), and have maintained a stable anti-diabetic drug regimen for morethan four months. Participants who were pregnant or breastfeeding, had cancer, consumed alcohol, smoked, or had a substance dependency, and those taking omega-3 fatty acid supplements or multivitamin-mineral supplements were excluded from the study.

### Intervention

Over a period of 12 weeks, the study’s subjects were categorized into four groups, taking into account their body mass index (BMI), the medication they received, and the amount prescribed. The stratified randomization technique was used to assign participants to two months of supplementation based on their sex and BMI. The four groups were as follows:

Group (1) Participants were given 2 capsules each containing 500 mg EPA and 200 mg DHA, as well as 1 capsule containing a nano curcumin placebo.

Group (2) Participants were given 1 capsule containing 80 mg nano curcumin and 2 capsules containing a placebo of ω − 3 Fatty Acids.

Group (3) Participants were given 2 capsules each containing 500 mg EPA and 200 mg DHA, as well as 1 capsule containing 80 mg nano curcumin.

Group (4) Participants were given 2 ω − 3 fatty acids placebos and 1 nano curcumin placebo (control group).

The capsule supplements used in the study were High EPAquatic® Omega 3 supplements and their placebos, which were made from oral paraffin and provided by Karen Pharma and Food Supplement Co. Each 1000 mg of High EPAquatic® contained 500 mg EPA and 200 mg DHA.

Additionally, the Nanocurcumin supplement used was Sinacurcumin® 80, with each 80 mg nano curcumin capsule comprising approximately 100% curcumin. The placebo for the Nanocurcumin supplement was made from oral paraffin and prepared by Exir Nano Sina Pharmaceutical Company.

### Outcomes

The primary outcome of was to determine the effect of EPA and DHA, and curcumin therapeutic supplements on anthropometric indicators, glucose homeostasis, and cardiometabolic risk markers, in DM2 patients. The secondary outcomes included the evaluation of the effect of the supplements on VEGF, lipid profiles, and inflammatory and oxidative stress indices. Additionally, we aimed to assess the safety and tolerability of the supplements, as well as the compliance of the participants with the supplement regimen.

The assessment of physical activity consisted of a brief survey that inquired about the length and strength of physical activity within the previous week. The grading system categorizes activities as intense, moderate, or light based on the amount of calories burned per minute. The physical activity questionnaire we used was a short form consisting of seven questions that evaluates a person’s physical activity over the past seven days. Vigorous activities require much physical strength and breathing harder than usual, while moderate activities require 3–6 calories per minute. Activities that are shorter than 10 min are excluded, and the overall energy intensity of activities from the previous week is determined using the guidelines established by The International Physical Activity Questionnaire (IPAQ). People are classified as weak, moderate, or intense physical activity based on their energy consumption.

The study measured the height and weight of participants using a Seca wall height meter with an accuracy of 1 mm. BMI was calculated using weight and height measurements, with a range of 18.5–24.9 kg/m^2^ considered average weight, 25–29.9 kg/m^2^ considered overweight, and 30 kg/m^2^ considered obese. WHO criteria were used to classify obesity [56]. Participants’ weight was measured throughout the study to evaluate their weighing state.

The measurement of plasma glucose levels was conducted through the use of a Pars Azmoon kit (manufactured in Iran) which utilizes the glucose oxidase/peroxidase method. This enzymatic and calorimetric method involves single-point measurement with a photometric method [57–59]. The measurement of serum insulin levels was conducted using The ELISA method and Monobind kit [60]. Insulin resistance was evaluated using the HOMA- IR index, which stands for homeostatic model assessment of insulin resistance [61]. Insulin resistance is indicated by a HOMA-IR value greater than 3.8 [62, 63]. The HOMA-IR index is calculated as follows:

$${\rm{HOMA}}-{\rm{IR}}=[{\rm{Fasting}}\,{\rm{Insulin}}\,{\rm{\mu }}{\rm{U}}/{\rm{ml}}* {\rm{Fasting}}\,{\rm{Glucose}}\,{\rm{mg}}/{\rm{dl}}]/405$$

To calculate insulin sensitivity, the QUICKI index (Quantitative Insulin Sensitivity Check) [43] was used, which is calculated as follows:

$${\rm{QUICKI}}=1/(\log ({\rm{fasting}}\,{\rm{insulin}}\,{\rm{\mu U}}/{\rm{mL}})\,+\,\log ({\rm{fasting}}\,{\rm{glucose}}\,{\rm{mg}}/{\rm{dL}}))$$

Lipid profile includes triglyceride, total cholesterol, HDL cholesterol, LDLD cholesterol, and VLDL cholesterol, measured using the Pars test kit with inter- and intra-assay coefficient variances below 5%.

### Adverse outcome

All the research staff instructed all the patients to notify them immediately if they experienced any health issues related to the study.

### Sample size

Using an effect size of Large according to Cohen’s criterion and G-power software, with a type 1 error of 0.05 and a test power of 80% in the two-sided test, a sample size of 100 people was estimated, with 25 people in each of the four groups.

### Randomization and blinding

Random allocation software was used to randomize the allocation of subjects to study groups, using 4 and 8 blocks with a specific 1-to-1 allocation ratio. To conceal the allocation, matching envelopes were used in a matte package numbered consecutively from 1 to 100. None of the participants, the researchers, or the statistical analysts knew the type of intervention received. During the study, weekly phone calls were made to ensure supplement consumption and prevent falling samples. At the end of every two weeks, participants were asked to bring their empty capsule shells to judge the correct consumption of the supplement. Returning the empty capsule shells as part of a meticulous adherence and treatment verification protocol. This measure was implemented to ensure that participants had not only received the assigned medication but had also followed the prescribed treatment regimen. Importantly, the empty capsule shells served as tangible evidence of medication consumption and facilitated a robust assessment of adherence. It’s crucial to note that the returned empty capsule shells were intentionally designed not to reveal any distinguishing features, preventing participants from discerning the specific groups to which they were assigned.

### Statistical analysis

The SPSS software version 18.0, developed by SPSS Inc. in Chicago, IL, was used to conductdata analysis. Normality of the data was inspected using the Kolmogorov–Smirnov test. Paired *t* tests were performed to compare variables within groups before and after standardization. Statistical differences among trial groups were assessed using the one-wayAnalysis of Variance (ANOVA) test. For data without normal distribution, two related sampletests (Wilcoxon) and K-independent sample tests (Kruskal–Wallis test) were used to compare variables before and after within groups and between groups, respectively. The two-way ANOVA with repeated measures plus Bonferroni’s correction was conducted to compare groups and evaluate the interaction of time*treatment (EPAquatic/Sinacurcumin) on all variables between the groups. Significance levels were determined as ηp2 (partial eta squared) ≥0.14 for significant findings, 0.06 to 0.14 for medium findings, and <0.06 for minor findings. The threshold for statistical significance was set at ≤0.05.

## Results

**Fig. 1:**
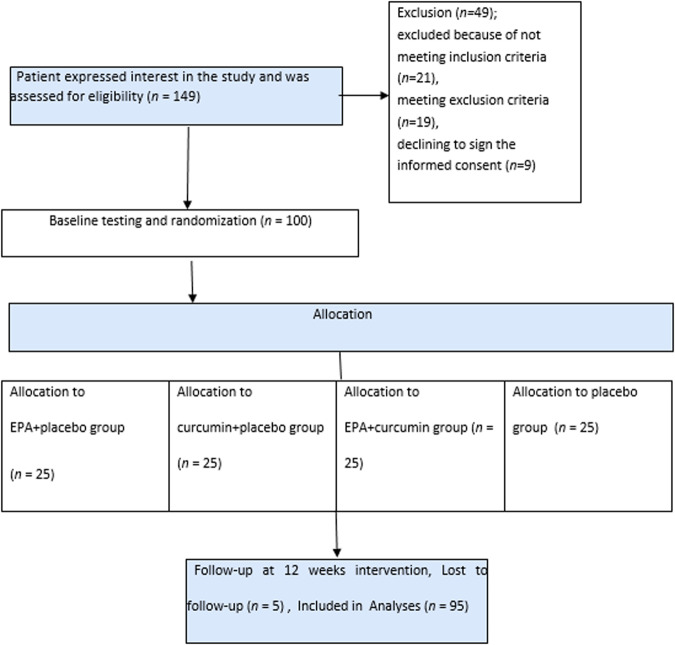
Flowchart of participants.

Recruitment of the first participant started on February 13th, 2021, and the last participant follow-up was on January 28th, 2022. Out of the 149 patients that were screened, 21 individuals were disqualified from the study for various reasons. Specifically, 21 did not meet the criteria for inclusion, 19 met the criteria for exclusion, and 9 declined to give informed consent. Out of the total of one hundred patients who were involved in the study, 95 of them successfully completed it (Fig. 1) The participants’ baseline and anthropometric characteristics were evenly distributed among the groups in the trial (Table 1). The mean age of the patients was 56.40 ± 9.55 years, and the mean BMI was 28.13 ± 2.15 kg/m^2^. 10% took Biguanides, 26% took Sulfonylureas, 2% took thiazolidinediones, 21% combined oral glucose-lowering drugs, and 22% injected insulin.

**Table 1:** Baseline general characteristics of the trial participants.

Variable		CP (*n* = 25)	EP (*n* = 25)	CE (*n* = 25)	PP (*n* = 25)	*p*
Age, Yrs.		54.56 ± 8.30	56.88 ± 8.36	56.68 ± 10.25	57.48 ± 11.27	0.727^b^
Sex	male	12 (48)	12 (48)	15 (60)	11 (44)	0.853^c^
	female	13 (52)	13 (52)	10 (40)	14 (56)	
Education	Illiterate	4 (16)	1 (4)	3 (12)	3 (12)	0.424^c^
	Primary School	12 (48)	13 (52)	16 (64)	13 (52)	
	High School	5 (20)	9 (36)	6 (24)	9 (36)	
	University	4 (16)	2 (8)	0 (0)	0 (0)	
Job	Unemployed	4 (16)	5 (20)	5 (20)	4 (16)	0.958^c^
	Housewife	7 (28)	5 (20)	7 (28)	7 (28)	
	Employed	11 (44)	10 (40)	9 (36)	9 (36)	
	Retired	3 (12)	5 (20)	4 (16)	5 (20)	
Smoking	Never	18 (72)	14 (56)	10 (40)	14 (56)	0.127^c^
	Former	4 (16)	8 (32)	7 (28)	6 (24)	
	Current	3 (12)	3 (12)	8 (32)	5 (20)	
Alcohol intake		10 (40)	3 (12)	6 (24)	4 (16)	0.093^c^
Duration of diabetes (Yrs)	<1	3 (12)	3 (12)	1 (4)	4 (16)	0.167^c^
	1–4	5 (20)	3 (12)	6 (24)	4 (16)	
	5–8	5 (20)	4 (16)	3 (12)	5 (20)	
	9–12	6 (24)	8 (32)	5 (20)	5 (20)	
	>12	6 (24)	7 (28)	10 (40)	7 (28)	
Family history of DM		17 (68)	18 (72)	20 (80)	21 (84)	0.551^c^
CVD		3 (12)	7 (28)	5 (20)	10 (40)	0.130^c^
Dyslipidemia		7 (28)	9 (36)	3 (12)	4 (16)	0.166^c^
HTN		7 (28)	7 (28)	8 (32)	7 (28)	0.986^c^
Antihypertensive drug	ACE inhibitors or ARBs	3 (12)	3 (12)	2 (8)	2 (8)	0.960^c^
	Diuretics	2 (8)	2 (8)	3 (12)	2 (8)	
	beta-Blockers	2 (8)	2 (8)	1 (4)	2 (8)	
	Calcium channel blockers	1 (4)	0 (0)	2 (8)	1 (4)	
Glucose-lowering medications	Biguanides	4 (16)	3 (12)	1 (4)	2 (8)	0.414^c^
	Sulfonylureas	5 (20)	10 (40)	6 (24)	5 (24)	
	SGLT2i	4 (16)	3 (12)	4 (14)	8 (28)	
	Thiazolidinediones	0 (0)	0 (0)	1 (4)	1 (4)	
	Combination BG-lowering drugs (oral)	6 (24)	6 (24)	5 (20)	4 (16)	
	Insulins/analogs Long-acting	1 (4)	0 (0)	2 (8)	2 (8)	
	Insulins/analogs Long-acting +Insulins/analogs Intermediate-acting	5 (20)	3 (12.)	6 (24)	3 (12)	
Cholesterol-lowering medications		13 (52)	12 (48)	15 (60)	13 (52)	0.860^c^
Weight, kg		72.42 ± 6.86	76.77 ± 6.18	76.12 ± 4.92	73.37 ± 6.76	0.064^ab^
BMI, kg/m^2a^		27.78 ± 2.15	28.90 ± 2.86	28.01 ± 1.52	27.82 ± 1.73	0.215^ab^
WC, cm		98.26 ± 6.22	100.78 ± 7.21	98.18 ± 4.70	97.93 ± 5.26	0.291^ab^
HC, cm		100.14 ± 10.78	103.81 ± 12.33	101.37 ± 7.72	101.58 ± 8.99	0.636^ab^
WHR		0.98 ± 0.06	0.97 ± 0.06	0.97 ± 0.04	0.96 ± 0.05	0.893^ab^
Dietary energy intake (kcal)		2305.54 ± 321.99	2404.59 ± 231.36	2412.20 ± 185.54	2387.58 ± 310.13	0.272^ab^
SBP (mmHg)		132.16 ± 21.33	141.12 ± 15.66	145.54 ± 18.97	131.48 ± 25.53	0.467^ab^
DBP (mmHg)		83.29 ± 14.11	91.21 ± 27.16	90.33 ± 25.14	87.33 ± 19.51	0.721^ab^

Data is presented as mean ± SD, or *n* (%), PP double placebo, CP curcumin plus placebo, EP EPA plus placebo, CE curcumin plus EPA, Kg kilogram, cm centimetre, % percent, Kj Kilojoule, METs Metabolic equivalents, SEM Standard error of the mean, IQR interquartile range.

^a^Body mass index is weight in kilograms divided by square of height in meters.

^b^Analysis of Variances (ANOVA).

^c^Kruskal–Wallis test.

When comparing dietary intakes before and after intervention, there was no significant difference observed in any of the groups. The mean serum concentrations of hs-CRP, TAC, and MDA did not show any statistically significant differences between the four groups at baseline. Table 2 presents the impact of supplements on inflammatory and oxidative stress indices.

**Table 2:** Serum concentrations of inflammatory and oxidative stress indices at baseline and post-intervention.

Variables	Time	CP (*n* = 24)	EP (*n* = 23)	CE (*n* = 25)	PP (*n* = 23)	Between-group difference in change, mean (95% CI)^b^					
						CP vs. EP	CP vs. CE	CP vs. PP	EP vs. CE	EP vs. PP	CE vs. PP
Hs-CRP	Before	9.30 ± 3.19	8.66 ± 2.09	9.42 ± 2.57	8.63 ± 1.45	0.50 (−0.34, 1.36)	0.28 (−0.56, 1.14)	−0.56 (−1.42, 0.28)	−0.21 (−1.07, 0.63)	−1.07 (−1.92, −0.21)	−0.85 (−1.70, −0.01)
	After	7.31 ± 2.21	6.94 ± 1.84	6.61 ± 2.58	9.11 ± 2.55						
	MD	−1.99 (−3.72, −0.26)	−1.71 (−2.89, −0.53)^a^	−2.84 (−4.51, −1.17)^a^	0.48 (−0.85, 1.82)						
TAC	Before	3.15 ± 1.41	2.91 ± 1.22	3.36 ± 1.27	3.24 ± 1.19	0.13 (−0.65, 0.38)	−0.72 (−1.24, −0.20)^a^	0.14 (−0.37, 0.66)	−0.85 (−1.37, −0.34)^a^	0.01 (−0.50, 0.52)	0.86 (0.35, 1.38)^a^
	After	3.54 ± 1.32	3.51 ± 1.39	4.78 ± 1.19	3.17 ± 1.22						
	MD	0.38 (−0.32, 1.09)	0.59 (−0.25, 1.45)	1.42 (0.69, 2.16)^a^	−0.07 (−0.69, 0.55)						
MDA	Before	6.00 ± 2.19	5.71 ± 1.99	6.91 ± 2.62	6.47 ± 1.77	−0.21 (−0.65, 1.08)	0.27 (−0.59, 1.14)	−0.62 (−1.48, 0.24)	0.06 (−0.80, 0.92)	−0.83 (−1.70, 0.03)	−0.89 (−1.76, −0.02)
	After	5.08 ± 2.93	5.25 ± 2.35	4.60 ± 2.01	6.28 ± 1.92						
	MD	−0.91 (−2.39, 0.56)	−0.45 (−1.84, 0.92)	−2.30 (−3.70, −0.89)^a^	−0.18 (−1.32, 0.95)						

^a^*p* < 0.01.

^b^obtain from two‐way repeated measures analysis of variance after adjusting for covariates.

The changes in serum concentrations of hs-CRP, TAC, and MDA showed a significant group*time interaction (*p* < 0.05). The partial eta squared (ηp2) of time and group*time interaction in hs-CRP (0.154 and 0.106) had significant effects. At the end of the trial, the serum concentration of hs-CRP reduced significantly in the EPA + Nano-curcumin (*p* < 0.01), EPA (*p* < 0.01), and Nano-curcumin groups (*p* < 0.05). There was a notable difference in changes in serum concentration of hs-CRP between the EPA and EPA + Nano-curcumin groups and the placebo group (*p* < 0.05).

ηp2 (partial eta squared) of time and group*time interaction and MDA (0.082 and 0.060) showed medium effects. After 12 weeks, the serum concentration of MDA reduced significantly within the EPA (*p* < 0.05), and EPA + Nano-curcumin groups (*p* < 0.01) decreased substantially in the EPA group (*p* < 0.01) In the study, it was found that the group taking EPA+ Nano-curcumin showed a noticeable contrast to the placebo group (*p* < 0.05).

Medium effects were observed for the partial eta squared (ηp2) of time and group*time interaction in TAC, with values of 0.101 and 0.088, respectively. The EPA + Nano-curcumin group showed a significant increase in serum concentration of TAC by the end of the trial (*p* < 0.01). In addition, there was a significant difference in changes in serum concentration of TAC between the EPA + Nano-curcumin and placebo groups (*p* < 0.01).

Table 3 shows the effects of using the EPA, Nano-curcumin, and EPA + Nano-curcumin supplements on participants’ weight. Participants’ weight changes in the trial groups did not show any significant group*time interaction at baseline or after 12 weeks of intervention (*p* > 0.05).

**Table 3:** The analysis of effects of intervention on weight at baseline and post-intervention.

		Mean ± SD							From baseline to 12 wk, mean (95% CI)
Variables		Baseline	2 wk	4 wk	6 wk	8 wk	10 wk	12 wk	Within-group change
weight	CP (*n* = 24)	72.42 ± 6.86	72.20 ± 6.31	72.21 ± 10.22	71.74 ± 6.71	70.69 ± 9.75	70.25 ± 12.40	70.41 ± 10.88	−2.01 (−7.47, 3.35)
	EP (*n* = 23)	76.77 ± 6.18	76.06 ± 5.65	76.21 ± 9.50	75.50 ± 6.86	74.13 ± 10.79	73.06 ± 10.31	73.17 ± 10.31	−3.59 (−8.24, 1.05)
	CE (*n* = 25)	76.12 ± 4.92	74.40 ± 5.22	73.80 ± 8.66	73.44 ± 7.74	73.89 ± 7.83	73.61 ± 6.29	74.04 ± 3.77	−2.08 (−6.16, 2.45)
	PP (*n* = 23)	73.37 ± 6.76	73.82 ± 8.37	72.89 ± 6.21	73.14 ± 7.00	72.92 ± 10.04	71.88 ± 5.68	72.32 ± 12.82	−1.05 (−6.91, 4.80)

Participants’ FBS changes in the trial groups did not show a significant group*time interaction at both baseline and after 12 weeks of intervention (*p* > 0.05). No meaningful changes concerning FBS were observed within the trial groups due to the treatments (all *p* > 0.05). Besides, FBS difference comparisons between the four groups were not significant (all *p* > 0.05).

Participants’ insulin concentration changes did not show any significant group*time interaction in the trial groups, both at baseline and after 12 weeks of intervention (*p* > 0.05). The patients who underwent a twelve-week supplementation with EPA + Nano-curcumin saw significant decreases in their insulin levels as reflected in their serum [MD: −1.44 (−2.70, −0.17)], which was significantly higher than changes in the placebo group [MD: −0.63 (−1.97, 0.69)].

Participants’ HOMA-IR changes did not show any significant group*time interaction in the trial groups, both at baseline and after 12 weeks of intervention (*p* > 0.05). Supplementation with the EPA [MD: −0.84 (−1.48, −0.19)], Nano-curcumin [MD: −0.75 (−1.38, −0.12)], and EPA +Nano-curcumin [MD: −1.00 (−1.71, −0.28)] caused statistically significant reductions of HOMA-IR index within groups. No meaningful change was observed in the control group. However, the HOMA-IR index difference between the four groups was insignificant (all *p* > 0.05).

At both baseline and after 12 weeks of intervention, there was no notable interaction between group and time regarding the changes in participants’ QUICKI (*p* > 0.05). Supplementation with EPA + Nano-curcumin [MD: 0.003 (0.000, 0.005)] caused a statistically significant increase in the QUICKI index within the group. However, the QUICKI index difference between the four groups was insignificant (all *p* > 0.05).

A noteworthy group*time interaction was detected for in HbA1c changes (*p* < 0.05). Supplementation with Nano-curcumin [MD: −0.17 (−0.33, −0.01)] and EPA + Nano-curcumin [MD: −0.22 (−0.34, −0.11)] caused statistically significant decreases of HbA1C within the groups. However, the HbA1C difference between the four groups was insignificant (all *p* > 0.05).

There was a noteworthy finding of group*time interaction in relation to alterations in serum TG (with a *p* value of less than 0.05). Significant effects were observed in ηp2 (partial eta squared) values for time and group*time interaction in TG, with values of 0.345 and 0.300, respectively. EPA [MD: −29.91 (−54.98, −4.83)] and EPA + Nano-curcumin [MD: −56.67 (−63.36, −49.98)] supplementations led to significant decreases in serum TG within the groups. There were approaching but not reaching statistical significance (*p* = 0.093) differences in TG serum levels between EPA + Nano-curcumin and placebo groups.

A noteworthy time effect was noticed for serum total cholesterol (*p* < 0.05). ηp2 (partial eta squared) of time in cholesterol (0.041) showed minor effects. EPA + Nano-curcumin [MD: −19.21 (−38.24, −0.18)] supplementations significantly decreased serum cholesterol within the groups. There were significant differences between Nano-curcumin and EPA groups [MD: −17.02 (−32.99, −1.05)] and also between Nano-curcumin and control group [MD: −20.76 (−36.73, −4.79)] concerning decreasing the serum cholesterol level.

A significant time effect was detected for serum LDL (*p* < 0.05). ηp2 (partial eta squared) of time in serum LDL (0.043) showed minor effects. EPA, Nano-curcumin, and EPA + Nano- curcumin supplementations resulted in non-significant decreases in LDL level (all *p* > 0.05) within the groups. However, LDL increased insignificantly in patients in the placebo group [MD: 4.56 (−25.30, 34.43)].

There were significant differences between Nano-curcumin and EPA groups [MD: −20.12 (−36.90, −3.34))] and also between Nano-curcumin and control groups [MD: −20.79 (−37.57, −4.01)] concerning decreasing the serum LDL level.

A significant time effect was detected for serum HDL (*p* < 0.05). ηp2 (partial eta squared) of time in serum HDL (0.072) showed medium effects. EPA + Nano-curcumin supplementation resulted in a notable rise of HDL [MD: (12.07 (4.05, 20.09)] within the group. The HDL level between EPA + Nano- curcumin and EPA groups showed a difference that was almost significant (*p* = 0.056).

A significant time effect and group*time interaction were detected for serum VEGF (*p* < 0.05). ηp2 (partial eta squared) of time and group*time in serum VEGF (0.075 and 0.150, respectively) showed significant effects. EPA [MD: 159.48 (23.08, 295.88)] and EPA + Nano- curcumin [MD: 377.14 (148.02, 606.26)] supplementations led to significant increases in serum VEGF (*p* < 0.05) within the groups. There were substantial differences between EPA and Nano-curcumin groups [MD: −127.50 376 (−247.91, −7.09)], between EPA and placebo groups [MD: 126.25 (5.83, 246.66)], between EPA + Nano-curcumin and Nano-curcumin groups [MD: −122.76 (−243.17, −2.35)] and between EPA + Nano-curcumin and placebo groups [MD: 121.50 (1.09, 241.92)]. Table 4 shows glycemic homeostasis indices, and markers of cardio-metabolic risk at baseline and post-intervention.

**Table 4:** Glycemic homeostasis indices, and markers of cardio-metabolic risk at baseline and post-intervention.

Variables	Time	CP (*n* = 24)	EP (*n* = 23)	CE (*n* = 25)	PP (*n* = 23)	Between-group difference in change, mean (95% CI)					
						CP vs. EP	CP vs. CE	CP vs. PP	EP vs. CE	EP vs. PP	CE vs. PP
FBS	Before	181.38 ± 38.25	183.14 ± 38.22	170.07 ± 31.39	186.71 ± 36.26	1.07 (−26.54, 28.69)	4.54 (−23.07, 32.16)	4.05 (−23.56, 31.67)	3.47 (−24.14, 31.09)	2.98 (−24.63, 30.60)	−0.48 (−28.10, 27.13)
	After	176.16 ± 41.84	176.85 ± 30.08	160.31 ± 36.24	177.43 ± 59.26						
	Within-group change	−0.86 (−14.68, 4.24)	−6.29 (−13.57, 0.99)	−9.76 (−19.86, 0.33)	−9.27 (−34.89, 16.33)						
Insulin	Before	14.75 ± 4.30	15.22 ± 4.31	13.55 ± 3.88	15.59 ± 4.07	−0.54 (−2.56, 1.48)	1.41 (−0.61, 3.43)	−1.03 (−3.05, 0.99)	1.95 (−0.07, 3.97)	−0.49 (−2.51, 1.53)	−2.44 (−4.46, −0.42)
	After	13.73 ± 3.80	14.35 ± 3.38	12.11 ± 3.59	14.96 ± 3.70						
	Within−group change	−1.02 (−2.09, 0.05)	−0.86 (−2.07, 0.33)	−1.44 (−2.70, −0.17)	−0.63 (−1.97, 0.69)						
HOMA-ir	Before	6.98 ± 3.39	7.24 ± 3.28	5.97 ± 2.84	7.53 ± 3.30	−0.21 (−2.30, 1.86)	1.13 (−0.95, 3.21)	−0.50 (−2.59, 1.57)	1.34 (−0.73, 3.43)	−0.29 (−2.37, 1.79)	−1.63 (−3.72, 0.44)
	After	6.22 ± 2.96	6.40 ± 2.22	4.97 ± 2.27	6.69 ± 2.95						
	MD	−0.75 (−1.38, −0.12)	−0.84 (−1.48, −0.19)	−1.00 (−1.71, −0.28)	−0.83 (−2.11, 0.43)						
QUICKI	Before	0.128 ± 0.009	0.127 ± 0.008	0.130 ± 0.008	0.126 ± 0.007	0.001 (−0.005, 0.007)	−0.003 (−0.009, 0.003)	0.001 (−0.005, 0.007)	−0.004 (−0.010, 0.002)	0.000 (−0.006, 0.006)	0.005 (−0.002, 0.011)
	After	0.129 ± 0.008	0.128 ± 0.006	0.133 ± 0.009	0.128 ± 0.008						
	MD	0.001 (−0.000, 0.003)	0.001 (−0.000, 0.002)	0.003 (0.000, 0.005)	0.002 (−0.000, 0.005)						
HbA1C	Before	9.06 ± 1.13	9.37 ± 1.17	9.14 ± 1.16	9.19 ± 1.41	−0.28 (−1.18, 0.61)	−0.05 (−0.95, 0.84)	−0.27 (−1.17, 0.63)	0.23 (−0.67, 1.13)	0.01 (−0.88, 0.91)	−0.21 (−1.11, 0.68)
	After	8.89 ± 1.03	9.15 ± 1.07	8.92 ± 1.20	9.30 ± 1.36						
	MD	−0.17 (−0.33, −0.01)	−0.22 (−0.45, 0.00)	−0.22 (−0.34, −0.11)	0.10 (−0.06, 0.27)						
TG	Before	178.02 ± 82.77	190.80 ± 77.35	174.00 ± 68.88	183.91 ± 82.87	−0.47 (−43.05, 42.11)	29.71 (−12.86, 72.30)	−6.63 (−49.21, 35.95)	30.18 (−12.39, 72.77)	−6.16 (−48.74, 36.42)	−36.35 (−78.93, 6.23)
	After	172.73 ± 73.67	160.89 ± 94.74	117.32 ± 60.39	180.11 ± 76.06						
	MC (95% CI)	−5.29 (−12.72, 2.13)	−29.91 (−54.98, −4.83)	−56.67 (−63.36, −49.98)	−3.80 (−10.31, 2.71)						
Chol total	Before	233.54 ± 31.20	243.38 ± 26.70	238.16 ± 29.36	235.56 ± 29.32	−17.02 (−32.99, −1.05)	−8.98 (−24.95, 6.98)	−20.76 (−36.73, −4.79)	8.04 (−7.93, 24.01)	−3.74 (−19.71, 12.22)	−11.78 (−27.75, 4.18)
	After	205.61 ± 73.30	229.81 ± 37.26	218.95 ± 44.28	245.12 ± 48.05						
	MD	−27.93 (−64.28, 8.41)	−13.56 (−31.12, 3.99)	−19.21 (−38.24, −0.18)	9.56 (−17.33, 36.46)						
LDL	Before	146.19 ± 31.47	157.42 ± 24.37	154.06 ± 31.11	149.97 ± 33.09	−20.12 (−36.90, −3.34)	−12.62 (−29.40, 4.15)	−20.79 (−37.57, −4.01)	7.49 (−9.28, 24.28)	−0.67 (−17.45, 16.11)	−8.16 (−24.95, 8.61)
	After	116.73 ± 78.94	145.75 ± 38.81	134.11 ± 45.94	154.54 ± 54.21						
	MD	−29.46 (−69.04, 10.11)	−11.66 (−27.71, 4.37)	−19.95 (−41.16, 1.24)	4.56 (−25.30, 34.43)						
HDL	Before	51.74 ± 15.43	47.80 ± 17.30	49.30 ± 12.68	48.80 ± 15.48	3.19 (−2.43, 8.83)	−2.30 (−7.93, 3.33)	1.35 (−4.28, 6.98)	−5.49 (−11.13, 0.13)	−1.84 (−7.47, 3.79)	3.65 (−1.98, 9.28)
	After	54.33 ± 16.71	51.88 ± 14.64	61.37 ± 14.10	54.56 ± 13.24						
	MD	2.58 (−6.32, 11.50)	4.08 (−6.23, 14.39)	12.07 (4.05, 20.09)^a^	5.76 (−3.79, 15.31)						
VEGF	Before	1431.95 ± 375.57	1529.86 ± 241.08	1416.28 ± 317.99	1552.37 ± 272.35	−127.50 (−247.91, −7.09)	−122.76 (−243.17, −2.35)	−1.25 (−121.66, 119.15)	4.74 (−115.67, 125.15)	126.25 (5.83, 246.66)	121.50 (1.09, 241.92)
	After	1532.24 ± 271.88	1689.34 ± 198.12	1793.43 ± 451.97	1414.33 ± 273.32						
	MD	100.28 (−88.18, 288.75)	159.48 (23.08, 295.88)	377.14 (148.02, 606.26)	−138.04 (−308.99, 32.91)						
Hs−CRP	Before	9.30 ± 3.19	8.66 ± 2.09	9.42 ± 2.57	8.63 ± 1.45	0.50 (−0.34, 1.36)	0.28 (−0.56, 1.14)	−0.56 (−1.42, 0.28)	−0.21 (−1.07, 0.63)	−1.07 (−1.92, −0.21)	−0.85 (−1.70, −0.01)
	After	7.31 ± 2.21	6.94 ± 1.84	6.61 ± 2.58	9.11 ± 2.55						
	MD	−1.99 (−3.72, −0.26)	−1.71 (−2.89, −0.53)^a^	−2.84 (−4.51, −1.17)^a^	0.48 (−0.85, 1.82)						
TAC	Before	3.15 ± 1.41	2.91 ± 1.22	3.36 ± 1.27	3.24 ± 1.19	0.13 (−0.65, 0.38)	−0.72 (−1.24, −0.20)^a^	0.14 (−0.37, 0.66)	−0.85 (−1.37, −0.34)^a^	0.01 (−0.50, 0.52)	0.86 (0.35, 1.38)^a^
	After	3.54 ± 1.32	3.51 ± 1.39	4.78 ± 1.19	3.17 ± 1.22						
	MD	0.38 (−0.32, 1.09)	0.59 (−0.25, 1.45)	1.42 (0.69, 2.16)^a^	−0.07 (−0.69, 0.55)						
MDA	Before	6.91 ± 2.62	6.00 ± 2.19	5.71 ± 1.99	6.47 ± 1.77	−0.21 (−0.65, 1.08)	0.27 (−0.59, 1.14)	−0.62 (−1.48, 0.24)	0.06 (−0.80, 0.92)	−0.83 (−1.70, 0.03)	−0.89 (−1.76, −0.02)
	After	4.60 ± 2.01	5.08 ± 2.93	5.25 ± 2.35	6.28 ± 1.92						
	MD	−2.30 (−3.70, −0.89)	−0.91 (−2.39, 0.56)^a^	−0.45 (−1.84, 0.92)	−0.18 (−1.32, 0.95)						

^a^*p* < 0.01.

### Safety and compliance

Adherence to the trial treatment (proportion of patients who took at least 75% of trial supplements) was more than 93%. Overall, 20.8% of participants receiving Nano− curcumin, 13.0% of participants receiving EPA, 12% of participants receiving EPA + Nano- curcumin, and The occurrence of adverse events in participants who received a placebo wasreported at 17.4%, and the difference between this group and the others was not found tobe statistically significant (*P* > 0.05). The adverse events that were reported were temporary and not significant. Common adverse events included flatulence, indigestion, constipation, and feeling nauseous.

## Discussion

The present study investigated the effects of EPA, Nano-curcumin, and EPA + Nano- curcumin supplements on oxidative stress, inflammation, and metabolic parameters in patients with diabetes. 100 patients were randomized, and 95 patients completed the trial. After 12 weeks of taking EPA + Nano-curcumin supplements, the patients experienced a statistically significant reduction in insulin levels in their blood. This decrease was significantly greater than the changes observed in the placebo group. The EPA + Nano- curcumin had a noteworthy decrease in hs-CRP levels compared to the placebo. Additionally, the EPA + Nano-curcumin experienced a substantial increase in TAC levels compared to the placebo.

However, there were no important dissimilarity in FBS, HOMA-IR index, QUICKI index, or HbA1C levels between the four groups. There were meaningful dissimilarity between the Nano-curcumin and EPA groups, and between the Nano-curcumin and control groups in terms of lowering the cholesterol level in serum. The Nano-curcumin group had notable decreases of LDL compared to the EPA group and the control group. The difference in TG serum levels between the EPA + Nano-curcumin and placebo groups was not statistically significant. There was a near-to-significant change of HDL between the EPA + Nano- curcumin and EPA groups. Finally, notable dissimilarities in the increase of VEGF in serum were there between the EPA and Nano-curcumin groups, the EPA and placebo groups, the EPA + Nano-curcumin and Nano-curcumin groups, and the EPA + Nano-curcumin and placebo groups.

Curcumin is renowned for its potent anti-inflammatory effects [64]. It modulates inflammatory pathways by inhibiting transcription factors activation such as Nuclear Factor-κB which is a potential method for the diagnosing and managing the Inflammatory CVD and reducing the interleukin-6 (IL-6) and tumor necrosis factor-alpha (TNF-α) expression, which are pro-inflammatory cytokines [64–66]. By doing so, curcumin helps mitigate chronic inflammation, which is a common feature of various chronic diseases, including DM2 [64, 67].

Curcumin possesses strong antioxidant properties [67]. It scavenges free radicals and ROS, thereby reducing oxidative stress [64]. Curcumin’s antioxidant capacity can help protect cells from damage caused by oxidative stress, which is implicated in the enhancement and advancement of DM2 and its related challenges [64, 67]. Curcumin may enhance insulin sensitivity by several mechanisms [67]. It may activate adenosine monophosphate-activated protein kinase (AMPK), a key regulator of energy metabolism [68, 69]. Activation of AMPK improves absorbing glucose and utilization in skeletal muscles [68]. Curcumin may have glucose-lowering effects through a potential mechanism of AMPK- mediated suppression of hepatic gluconeogenesis [68, 69]. Curcumin can also modulate insulin receptor signaling pathways, potentially increasing insulin responsiveness [70, 71]. Curcumin has been shown to protect pancreatic beta-cells, which are responsible for insulin production [72]. It may help preserve beta-cell function and prevent their deterioration, a crucial factor in the development of DM2 [72]. Curcumin can influence metabolism of lipids by decreasing the gene expressions which are involved in synthesizing cholesterol and escalating the gene expressions which are related to cholesterol efflux [67, 73]. This modulation may contribute to its lipid-lowering effects.

EPA, an omega-3 fatty acid primarily originates from fatty fish, exerts several beneficial effects on health. The EPA is widely recognized for its ability to reduce inflammation [24, 26]. It competes with arachidonic acid, an omega-6 fatty acid, in the synthesis of eicosanoids [46, 74]. This competition results in a shift towards the production of less inflammatory eicosanoids [74]. The production of pro-inflammatory cytokines can be decreased by EPA, helping to quell chronic inflammation [24, 26, 75]. EPA has been shown to enhance insulin sensitivity by promoting the uptake of glucose in muscle cells and by modulating insulin receptor signaling pathways [76]. These effects contribute to improved glycemic control, making EPA an attractive option for individuals with DM2. EPA can help reduce the risk of cardiovascular complications by lowering TG levels, decreasing blood pressure, and reducing the formation of blood clots [76, 77]. It may also improve endothelial function, which is vital for healthy blood vessel function [76, 77]. EPA, like other omega-3 fatty acids, possesses antioxidant properties that protect cells from oxidative damage [24, 26]. This effect can help reduce oxidative stress in individuals with DM2. EPA can reduce triglyceride levels, which are often elevated in individuals with DM2. It may also lead to modest reductions in LDL cholesterol levels, contributing to better lipid profiles [74, 76]. When curcumin and EPA are combined, they may exhibit synergistic effects in managing DM2 or any other metabolic pathways [71, 78]. The dual impact on insulin sensitivity from curcumin and EPA may be complementary, potentially leading to more substantial improvements. Curcumin’s influence on insulin signaling pathways, along with EPA’s ability to enhance glucose uptake, can work together to improve glycemic control in individuals with DM2 [71]. The observed impacts of EPA and nano-curcumin on oxidative stress, insulin sensitivity, inflammation, and lipid profiles may be attributed to their individual and synergistic mechanisms [71, 78]. EPA’s anti-inflammatory properties, along with its potential to modulate lipid metabolism and oxidative stress, may contribute to the observed improvements [75, 79]. Curcumin’s antioxidant and anti-inflammatory effects are well- documented and may complement EPA’s actions. The combination of these two compounds could result in enhanced efficacy, addressing multiple facets of DM2 pathophysiology [71, 78].

These findings have noteworthy clinical implications for the management of DM2. The combination of EPA and Nano-curcumin shows promise as a multifaceted approach to improving insulin sensitivity, reducing inflammation, and enhancing antioxidant capacity in individuals with DM2. Additionally, the modulation of lipid profiles and improvements in vascular health underscore the potential for mitigating cardiovascular risk—a crucial consideration in DM2 management [71, 75, 78].

Neerati et al. [73] investigated the potential benefits of curcumin in reducing lipids and inhibiting permeability glycoprotein, and its effect on the way glyburide is processed and its impact on patients with DM2 diabetes mellitus. They observed changes in the patients’ glyburide concentrations, glucose levels, and lipid profiles. The results showed a notable reduction in LDL and TG and increased HDL content. They suggested that taking curcumin capsules alongside glyburide could improve glycaemic control and that curcumin may have potential as a drug with lipid-lowering and anti-diabetic properties [73]. While their study provided valuable insights into the specific effects of curcumin in conjunction with glyburide, it is important to emphasize that our present investigation does not directly assess the interaction between curcumin and anti-diabetic drugs. Future research should consider more targeted investigations, such as exploring the specific interactions between curcumin and distinct anti-diabetic medications, which could further refine treatment approaches and advance personalized strategies for managing DM2. This nuanced understanding, fosters a forward-looking perspective that encourages ongoing exploration and innovation in the field of diabetes therapeutics.

Hodaei et al. [80], studied the impact of curcumin supplementation on DM2 was investigated for 10 weeks. The results presented that compared to a placebo, supplementation with curcumin caused significant changes in mean weight, BMI, and FBS. However, HbA1c, insulin, MDA, TAC, HOMA-IR, and pancreatic B cell function (HOMA-B) did not show any meaningful changes when the study concluded [80].

In a trial conducted by ref. [81], the impact of omega 3 PUFA (DHA + EPA) on adipokines, metabolic control, and lipid profile in DM2 patients was analyzed. The group receiving omega 3 PUFA showed significant reductions in blood glucose levels. After 24 weeks, the omega 3 PUFA and control groups showed significant decreases in Hb1Ac, leptin, and leptin to adiponectin ratio. However, a substantial increase in serum resistin, insulin, and HOMA-IR in both groups were shown. Omega 3 PUFA supplementation resulted in an overall enhancement in lipid profile with a notable reduction in TG and atherogenic index.

Conversely, the control group experienced increased total cholesterol, non-HDL cholesterol, and atherogenic index [81].

Thota et al.[71], investigated the effects of curcumin and long-chain omega-3 PUFA (mainly DHA, but also EPA) supplementation on patients at high risk of developing DM2 were evaluated. Participants were given either a double placebo, curcumin plus placebo matching for omega-3 PUFA, omega-3 PUFA plus placebo matching for curcumin, or curcumin plus omega-3 PUFA for 12 weeks. After the trial, HbA1c and fasting glucose levels stayed unaffected across all groups, but insulin sensitivity showed meaningfully improvement in the group that received curcumin supplementation compared to the placebo. The groups which received omega-3 PUFA and curcumin+ omega-3 PUFA tended to improve insulin sensitivity, but the difference was insignificant. TG levels increased in the placebo group, but curcumin and curcumin+ omega-3 PUFA supplementation reduced TG levels, with omega-3 PUFA resulting in the most significant reduction. Reducing insulin resistance and TG levels with curcumin and omega-3 PUFA could effectively reduce the risk of developing DM2. However, the study did not demonstrate the complementary benefits of these supplements on glycaemic control [71].

Differences in study design, including intervention dosage, duration, patient population, and outcome measures, can impact the inconsistent findings on the impact of curcumin or EPA on insulin concentration, inflammation, and metabolic indexes. Confounding factors like dietary habits, exercise, and medication use, as well as biological variability, can also contribute to varying results. Careful patient selection and consideration of individual differences are crucial to obtaining accurate outcomes. It is essential to acknowledge certain limitations of our study, including the quite short period of the intervention period and its small sample size. Longer-term studies are necessary to assess the sustainability of the detected effects and their impact on long- term glycemic control and cardiovascular outcomes. Moreover, exploring the specific molecular mechanisms underlying these effects and potential dose-response relationships could further enhance our understanding of these interventions.

## Conclusion

In conclusion, this study offers compelling indication of the prospective benefits of EPA, Nano-curcumin, and their combination in improving insulin sensitivity, reducing inflammation, modulating lipid profiles, and enhancing vascular health in individuals with DM2. While some parameters of glycemic control remained stable over the 12-week period, the observed changes in insulin levels and other metabolic markers underscore the promise of these interventions. Future research should delve deeper into the mechanisms involved and consider longer-term outcomes to substantiate these findings and inform clinical practice in DM2 and its complications management.

## Data Availability

The data that support the findings of the present study are available upon reasonable request from the corresponding author. All relevant data generated and analyzed during the study, including clinical measurements, paraclinical assessments, and statistical analyses, will be made available to qualified researchers who wish to replicate or verify the results presented in the published manuscript.
